# Specialized Feed-Additive Blends of Short- and Medium-Chain Fatty Acids Improve Sow and Pig Performance During Nursery and Post-Weaning Phase

**DOI:** 10.3390/ani14243692

**Published:** 2024-12-20

**Authors:** Sandra Villagómez-Estrada, Diego Melo-Durán, Sandra van Kuijk, José F. Pérez, David Solà-Oriol

**Affiliations:** 1 Animal Nutrition and Welfare Service (SNIBA), Department of Animal and Food Science, Universitat Autonòma de Barcelona, 08193 Bellaterra, Spain; diego.melo@ute.edu.ec (D.M.-D.); josefrancisco.perez@uab.cat (J.F.P.); david.sola@uab.cat (D.S.-O.); 2 Faculty of Veterinary Medicine and Agronomy, Universidad UTE, Quito 17012764, Ecuador; 3 Trouw Nutrition, Research and Development Department, Stationsstraat 77, 3811 MH Amersfoort, The Netherlands; sandra.van.kuijk@trouwnutrition.com

**Keywords:** organic acids, short-chain fatty acids, nursery sows

## Abstract

The present study investigates the impact of supplementing diets with a synergistic blend of short- and medium-chain fatty acids (SCFAs-MCFAs) during the peripartum and lactation phases on early microbial colonization and the subsequent growth performance of newborn pigs. The experiment involved 72 sows and their litters, with a follow-up on 528 weaned pigs. Sows were fed either a control diet or a diet supplemented with SCFAs-MCFAs and the pigs were monitored for their growth performance and microbial populations. Subsequently, selected weaned pigs were allotted to an SCFAs-MCFAs diet according to the maternal diet. Results showed that SCFAs-MCFAs supplementation led to reduced backfat loss in sows and improved pig weight and uniformity at weaning (*p* < 0.05). Additionally, suckling pigs exhibited significant shifts in gut microbiota, including increased lactic acid bacteria and reduced *Streptococcus suis* populations (*p* < 0.05). Although there was no influence of maternal diet on pig growth after weaning, there was a modulation on bacterial populations at 7 and 35 days post-weaning. Pigs fed SCFAs-MCFAs demonstrated improved feed efficiency with notable reductions in *E. coli* and *Streptococcus suis* counts. The findings suggest that maternal dietary supplementation with SCFAs-MCFAs can positively influence both sow and pig performance, offering a potential strategy to enhance productivity and health in the commercial swine production.

## Simple Summary

This study explores the effects of adding specific organic acids to the diets of sows during late pregnancy and nursing, and how this affects the growth and health of their piglets. The research involved 72 sows and their litters, tracking 528 piglets after weaning. The sows were given either a standard diet or one enhanced with organic acids, and their piglets were monitored for growth and microbial counts. The results showed that the supplemented diet helped to minimize fat loss in sows and led to heavier, more uniform piglets at weaning. It also altered the gut bacteria of pigs, increasing beneficial bacteria and reducing harmful ones like *E. coli*. While the maternal diet did not significantly affect pig growth after weaning, it did continue to influence their gut health. These findings suggest that enhancing sow diets with specific organic acids can improve both the health of the mothers and the growth and well-being of their piglets, which could be a valuable approach for boosting productivity in pig farming.

## 1. Introduction

Weaning is recognized as a highly stressful period for young pigs, during which they experience maternal separation, new social and dietary conditions, and critical physiological changes. These stressors, often compounded by suboptimal sanitary conditions on farms, can lead to severe outbreaks of diarrhea, typically linked to the proliferation of specific pathogens. The most commonly implicated bacterial pathogen is *Escherichia coli* [1]. However, once dysbiosis and intestinal disruption begin, other pathogenic bacteria, such as *Clostridium* spp., *Streptococcus suis*, and *Salmonella* spp., can rapidly proliferate. Notably, the high incidence of *S. suis* can result in severe clinical manifestations, including meningitis, arthritis, and sudden death in post-weaning pigs [2].

A strategy that has been proposed to mitigate these weaning challenges is to promote early colonization of newborn and suckling pigs with beneficial saprophytic bacteria, such as lactic acid bacteria. The pig gut is rapidly colonized by various microorganisms during and after birth, primarily transferred from the sow (e.g., through the birth canal and skin) and the surrounding farrowing environment (e.g., feces, urine). Early-life microbial exposure is crucial for growth, immune system development, and long-term health [3]. Disruptions in intestinal microbial composition during the neonatal period can lead to the growth of undesirable bacterial communities and induce a proinflammatory state [4]. Therefore, optimizing maternal nutrition to influence early gut microbiota establishment in piglets is a promising strategy to prevent intestinal disorders at weaning and promote health and growth. Following the ban on antibiotic growth promoters, various feed additives, such as essential oils, plant-derived compounds, and organic acids, among others, have been explored as functional tools to manage gastrointestinal dysbiosis and support gut health. Thus, in commercial practice, organic acids (OAs) and their salts, primarily in the form of short-chain fatty acids (SCFAs) and medium-chain fatty acids (MCFAs), are widely utilized due to their proven efficacy in addressing these challenges. Multiple studies have highlighted their positive effects on preventing pathogen colonization, enhancing digestive function, supporting the immune system, and improving overall growth pig performance, particularly in modern pig production system [5,6]. The main mechanisms of action of SCFAs and MCFAs are linked to a broad spectrum of direct and indirect benefits for animal health, production, and nutrient digestibility [5], complemented by their documented antiviral and antibacterial properties [7,8]. SCFAs are monocarboxylic fatty acids with up to four carbon atoms, including their salts (e.g., acetate, propionate, and butyrate). Physiologically, these compounds are end products of gut microbial fermentation of indigestible carbohydrates, such as fiber and resistant starch, predominantly by anaerobic populations in the large intestine [9]. MCFAs, on the other hand, are monocarboxylic fatty acids containing 6 to 12 carbon atoms (e.g., caproic, caprylic, capric, lauric acids) and are naturally found in milk fat and vegetable oils, such as coconut and palm kernel oil. The combination of SCFAs and MCFAs has demonstrated synergistic benefits for pig health and growth performance [10,11], attributed to their differing pKa values and broad-spectrum antimicrobial activity [5]. This combination has provided a strategic approach to improving gut health and supporting overall production efficiency in post-weaning and growing pigs. However, the potential of incorporating organic acids into sow diets to modulate intestinal microbial composition and, in turn, enhance the health and performance of both suckling and post-weaning piglets, has received limited attention and warrants further investigation [11,12,13]. Some promising studies have highlighted that supplementing sow diets with sodium butyrate (SCFAs) and MCFAs can positively impact both sow reproductive performance and piglet health. These benefits include a shortened weaning-to-estrus interval, a reduction in the incidence of diarrhea in suckling piglets, and increased concentrations of fat, protein, and immunoglobulins (IgA, IgG, IgM) in colostrum [14,15], which are crucial for supporting piglet immunity and early growth.

In the present study, we hypothesize that supplementing sow diets with a specialized blend of SCFAs and MCFAs during the peripartum and lactation periods will stimulate the proliferation of beneficial saprophytic bacteria in the sows, thereby positively influencing the early microbial colonization of newborn piglets during the lactation phase. This modulation is expected to enhance intestinal microbial structure and therefore improve feed efficiency during both pre- and post-weaning phases. Furthermore, continued supplementation of SCFAs and MCFAs in post-weaning diets is anticipated to aid in the control of intestinal pathogens, contributing to improved growth performance and overall health in weaned pigs.

## 2. Materials and Methods

### 2.1. Sow Experiment

#### 2.1.1. Animals and Housing

At the end of late gestation (110 d post-mating), a total of 72 Landrace × Large White sows (ranging from nulliparous to 8th parity), from the same commercial farm, were individually weighed and assigned to one of two experimental diets (*n* = 36 per diet). Sows were housed in individual farrowing crates (4.68 m^2^) with fully slatted floors for the sows and heated hard plastic flooring for the piglets. Each farrowing pen was equipped with a sow feeder and two nipple drinkers (one for the sow and one for the piglets) to ensure unrestricted access to feed and water. The farrowing facility was environmentally controlled, with natural daylight provided through windows and supplemental artificial lighting programmed as needed. Ventilation was managed with single, variable-speed fans linked to temperature sensors and cooling mechanisms. After weaning, the sows were kept in individual crates until they reached estrus. Piglets were weaned 21 days after farrowing and then transferred to the nursery unit within the farm to continue with the post-weaning phase of the experiment.

#### 2.1.2. Experimental Design and Dietary Treatments

During the gestation phase, all sows were fed the same commercial feed without distinction. When the sows were transferred to the farrowing crates prior to parturition (110 days post-mating), they were provided with their assigned experimental lactation diet. A basal diet (Table 1) was formulated to meet or exceed the nutrient requirements as outlined by the NRC [16]. The sows were assigned to one of two experimental diets: a maternal control diet (MCON); the same control diet supplemented with a short- and medium-chain fatty acid mixture (SCFAs-MCFAs) consisting of sorbic acid, formic acid, acetic acid, lactic acid, propionic acid, and a mixture of C-8 to C-12 MCFAs (Selacid Green Growth, Tilburg, the Netherlands) at a concentration of 3 kg/ton of complete feed (MOA). On the day of farrowing, sows were not fed, and starting the following day, the feed amount was gradually increased by 1.0 kg daily until ad libitum feeding was reached and maintained until weaning.

#### 2.1.3. Experimental Procedures and Sampling

Sow body weight (BW) and backfat thickness were recorded at day 110 post-mating and at weaning (21 days post-farrowing). Backfat thickness was measured using a portable veterinary ultrasound scanner (model WED-3000 V, Welld, Shenzhen, China) at the P2 position (7.5 cm from the midline at the last rib). Sow feeders (Rotecna, Lleida, Spain) were manually filled twice daily (at 8:00 and 16:00) to ensure ad libitum intake. Daily individual feed intake was monitored by weighing the feed offered and the refusals, which were collected and weighed the next morning before the subsequent feeding.

Reproductive performance measurements included the total number of piglets born (alive, stillborn, and mummified), as well as individual piglet BW at birth, during cross-fostering, and at weaning. Within 24–48 h after birth, piglets were identified with ear tags and injected with 200 mg of iron (iron dextran). Cross-fostering was allowed within the same experimental diet group up to 24 h post-farrowing, maintaining a standard of 12 piglets per sow. Piglets were individually weighed on day 20 post-farrowing, and no creep feed was provided to avoid interference with post-weaning results. The interval from weaning to estrus (weaning-to-estrus interval; WOI) was also recorded. Neither sows nor litters received antibiotics during the experimental period or the preceding gestation.

A subset of 24 sows per diet group was selected based on parity (MCON = 3.5; MOA = 3.4) for sampling. Fecal samples from sows were collected via rectal stimulation at day 110 post-mating (start of the trial), day 7 post-farrowing, and at weaning. Similarly, fecal samples were collected from three piglets per litter, chosen from the same subset of sows, at days 7 and 21 post-birth using sterile swabs (Deltalab, Barcelona, Spain).

### 2.2. Pig Experiment

#### 2.2.1. Animals and Housing

At weaning, 528 pigs ([Landrace × Large White] × Pietrain), selected from the previously described sow experiment and with an initial BW of 6.2 ± 0.64 kg, were used in a 35 d study. Pigs were individually weighed and blocked according to maternal diet and initial BW, then distributed into four experimental groups housed in 48 pens (12 pens per diet group, with 11 pigs per pen). Male and female pigs were randomly assigned to the same pens. Each pen, measuring 3.12 m^2^, featured a fully slatted floor, was equipped with a commercial non-lidded feeder (TR5, Rotecna, Lleida, Spain) and a nipple drinker to ensure ad libitum access to feed and water. The facility was equipped with environmental controls, including thermostatically regulated heaters and exhaust fans, to maintain optimal temperature and ventilation. These settings were adjusted before housing the newly weaned pigs. Feed was provided ad libitum throughout the duration of the study.

#### 2.2.2. Experimental Design and Dietary Treatments

The two-phase diets (Table 2) were formulated to meet or exceed the nutrient requirements outlined by the NRC [16], with a pre-starter (PS) phase from day 1 to 14 and a starter (ST) phase from day 15 to 35. The study was designed as a 2 × 2 factorial arrangement within a split-plot design, incorporating two experimental lactation diets for sows (without SCFAs-MCFAs, MCON; or with SCFAs-MCFAs, MOA) and two post-weaning diets for pigs (without SCFAs-MCFAs, CON; or with SCFAs-MCFAs, OA). Consequently, two post-weaning diets were formulated: a control diet (CON); a control diet supplemented with a blend of SCFAs and MCFAs (OA). The SCFAs-MCFAs supplementation consisted of a specialized blend of organic acids with high pKa, MCFAs, slow-release lauric acid, target-release butyrates, a phenolic compound (Presan FX, Tilburg, The Netherlands) at 2 kg/ton, and an organic acid mixture consisting of sorbic acid, formic acid, acetic acid, lactic acid, and propionic acid (Fysal MP, Tilburg, the Netherlands) at 6 kg/ton in the pre-starter diet. For the starter diet, the supplementation included 2 kg/ton of the synergistic blend (Presan FX) and 4 kg/ton of the organic acid mixture (Fysal MP). All diets were provided ad libitum in pellet form. In both the sow and pig experiments, SCFAs-MCFAs products were added on top of the formulated feed before pelleting.

Composite feed samples (1 kg) were collected for each experimental diet during the bagging process and subsequently divided into four 250 g portions for future analysis. The diets did not include zinc oxide at pharmacological levels, nor were antibiotics or other antimicrobial feed additives incorporated.

#### 2.2.3. Experimental Procedures and Sampling

The BWs of each pig and the remaining feed in the feeders were recorded on days 14 and 35. These data were used to calculate average daily gain (ADG), average daily feed intake (ADFI), and the gain-to-feed (G:F) ratio. On day 7 post-weaning, fecal samples were collected from three pigs per pen, selected based on the median BW within the pen, using sterile swabs. Fecal samples were collected from the same pigs again on day 35 post-weaning. All samples were immediately stored at 4 °C until processing and analysis. The pigs selected for fecal sampling were different from those used in the sow experiment.

### 2.3. Chemical Analysis

Analytical determinations of diets were performed according to the AOAC International [17] methods for dry matter (Method 934.01), Dumas method for crude protein (Method 968.06), and the traditional Soxhlet extraction method for ether extract (Method 920.39) and ash (Method 942.05). Neutral detergent fiber was analyzed using the Ankom nylon bag technique (Ankom 200 fiber Analyzer, Ankom Technology, Macedon, NY, USA).

### 2.4. Microbial Count

All fecal samples were analyzed for *Escherichia coli*, *Streptococcus suis*, *Clostridium perfringens*, and lactic acid bacteria via plating. Briefly, *E. coli* counts were carried out using MacConkey agar (Oxoid, Lyon, France) incubated at 37 °C under aerobic conditions for 24 to 48 h. *Streptococcus suis* was plated in Blood agar (Becton Dickinson, Grenoble, France) and incubated at 37 °C with 5% CO_2_ for 24 to 48 h. *Clostridium perfringens* were determined using TSN agar (Biokar, Allonne, France) incubated at 42 °C under anaerobic conditions for 24 to 48 h. Finally, lactic acid bacteria counts were determined using MRS agar (Biokar, Saint-Eustache, QC, Canada) incubated at 37 °C with 5% CO_2_ for 24 to 48 h.

### 2.5. Statistical Analysis

Data were analyzed using ANOVA with the MIXED procedure of SAS (version 9.4, SAS Institute, Cary, NC, USA). The individual sow was considered the experimental unit for sow performance parameters. The statistical model included the experimental diet as a fixed effect and parity group as a random effect. The sows were blocked into three parity groups (0 to 2nd parity, 3rd to 4th parity, and 5th to 8th parity). Reproductive performance parameters included the total number of piglets born, the number born alive, stillborn piglets, and the weight of the live litter, as well as the average weight of the live piglets. Cross-fostering piglet weight was used as a covariate for analyzing reproductive performance at weaning.

Post-weaning pig data were analyzed using a 2 × 2 factorial arrangement. The statistical model included the fixed effects of maternal diet, pig diet, and the interaction between maternal and pig diets. Block was considered a random effect, and pen was treated as the experimental unit for performance responses. If the two-way interaction between sow and pig diet was found to be non-significant, it was removed from the model, and the data were reanalyzed for the main effects. Consequently, the main effects are discussed for responses where the interaction was not significant.

Microbial counts were log-transformed prior to analysis. Initial sow microbiota counts were used as covariates for subsequent measurements (days 7 and 20) using the MIXED procedure. Pig microbiota during the lactation and nursery phases was analyzed using the MIXED procedure, with the individual pig considered the experimental unit.

The normality and homogeneity of variance were assessed using the Shapiro–Wilk test. Means that showed significant differences were separated using Tukey’s adjustment. Statistical significance was determined at *p* ≤ 0.05, and tendencies were noted for *p*-values between >0.05 and <0.10.

## 3. Results

### 3.1. Sow Experiment

#### 3.1.1. Sow Reproductive Performance

The productive performance response of sows in the experimental diets is presented in Table 3. SCFAs-MCFAs supplementation did not significantly affect sow feed intake, body weight, or backfat thickness at weaning, except for backfat loss (*p* = 0.023). Sows fed the control diet experienced greater backfat loss compared to those on the organic acid-supplemented diet (−3.73 mm vs. −2.88 mm) during the lactation period.

As expected, no differences between experimental diets were observed on sow reproductive performance at farrowing (*p* > 0.10; Table 4). At the end of suckling period, litter weight or number of weaned pigs did not differ between experimental groups; however, pigs nursed by sows fed organic acid diet were 320 g heavier than those from the control diet (*p* = 0.027). Likewise, the influence of organic acid supplementation was observed on reduction in the BW coefficient of variation within the litter (−2.68%; *p* = 0.024).

#### 3.1.2. Microbial Counts

**Figure 1:**
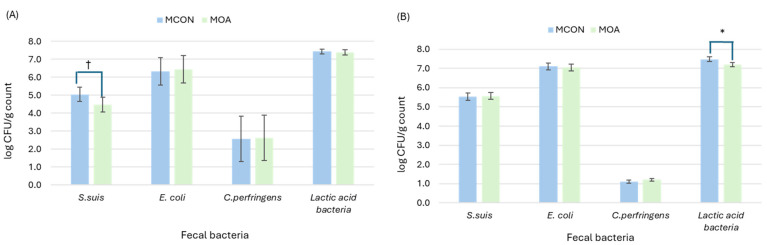
Plating microbial count from lactating sows (**A**) and suckling pigs (**B**) as affected by sows fed control (MCON) or SCFAs-MCFAs supplementation (MOA) diet at 7 d after farrowing. Data from sows were analyzed with microbial counts at 0 d as covariable. Data from suckling pigs is mean of 3 samples from 24 L per experimental diet. Asterisk (*) and cross (†) symbol indicates significant differences (*p* = 0.020) and tendencies (*p* = 0.083), respectively.

**Figure 2:**
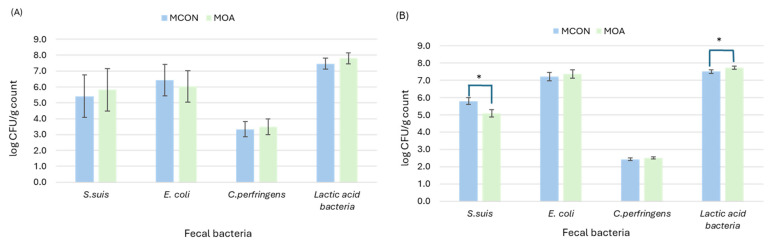
Plating microbial count from lactating sows (**A**) and suckling pigs (**B**) as affected by sows fed control (MCON) or SCFAs-MCFAs supplementation (MOA) diet at 20 d after farrowing. Data from sows were analyzed with microbial counts at 0 d as covariable. Data from suckling pigs are means of 3 samples from 24 L per experimental diet. Asterisk (*) symbol indicates significant differences (*p* < 0.05).

The results of the microbial count of sows and suckling pigs are presented in Figure 1 and Figure 2. Sow microbial counts at the start of experiment were used as covariable to successive sow measures at d 7 and 20 post-farrowing. At day 7, feeding sow organic acids during lactation had no effect on Escherichia coli, Clostridium perfringens, nor lactic acid bacteria counts. *Streptococcus suis* counts tended to decrease (4.48 vs. 5.05 log CFU count; *p* = 0.083). In contrast, in suckling pigs, lactic acid bacteria counts decreased when organic acids were fed to sows (7.20 vs. 7.48 log CFU count; *p* = 0.020). No differences in *S. suis*, *E. coli* and *C. perfringens* counts were observed (*p* > 0.10). At the end of the suckling period, although no differences in sow microbiota profile were observed, suckling pigs had a decreased count of *S. suis* (5.09 vs. 5.81 log CFU count; *p* = 0.001) and an increased count of lactic acid bacteria (7.73 vs. 7.51 log CFU count; *p* = 0.019).

### 3.2. Pig Experiment

#### 3.2.1. Pig Growth Performance

There was no effect of the interaction between maternal and pig diet for any of the growth performance responses (*p* > 0.10). Therefore, the data were reanalyzed and only the main effects of maternal and pig diet are presented in Table 5. The organic acid supplementation on sow lactating diet was not observed to have any effect on the pig growth performance during the post-weaning experimental periods (*p* > 0.10). At the end of pre-starter phase, pigs fed the organic acid supplementation had a higher G:F than those fed the control diet (*p* = 0.050). Likewise, the organic acid supplementation tended to improve the BW at the end of the pre-starter (*p* = 0.099) and starter phases (*p* = 0.068), increasing the final BW by 350 g. Overall, the mortality rate was 1.14% and was not related to any experimental diet (*p* > 0.10).

#### 3.2.2. Microbial Counts

During post-weaning performance, a two-way interaction between sow and pig diet was observed for *C. perfringens* and lactic acid bacteria on day 7 and for *E. coli* on day 35 (*p* < 0.05; Table 6). Pigs that were nursed by sows fed organic acid diet and that were fed with control diet post-weaning had the lowest *C. perfringens* counts 7 days after weaning. Pigs nursed by control sows that were fed the OA diet post-weaning had the lowest lactic acid bacteria counts (*p* = 0.008). At the end of starter period, pigs nursed by control sows and that were fed control diet post-weaning showed higher *E. coli* fecal counts than pigs nursed by control sows but fed the organic acid diet (7.21 vs. 6.66 log CFU count; *p* = 0.002). The influence of maternal diet was noted until 7 days post-weaning, where pigs nursed by MOA sows had lower *C. perfringens* (3.47 vs. 3.63 log) but higher lactic acid bacteria (8.02 vs. 7.93 log) counts than those nursed by MCON sows (*p* > 0.05). Taking pig diet separately, OA diets reduced the counts of *S. suis* (2.97 vs. 3.31 log), E.coli (7.48 vs. 7.89 log), and lactic acid bacteria (7.92 vs. 8.03 log), while increasing *C. perfringens* (3.61 vs. 3.49 log) after seven days of feeding. At 35 days post-weaning a reduction on *E. coli* (6.81 vs. 7.04 log) but increased *C. perfringens* counts (3.32 vs. 3.19 log).

## 4. Discussion

In commercial swine practice, one of the primary concerns following the birth of piglets is ensuring their survival and optimal development. In the present study, results indicated that supplementing maternal diets with short- and medium-chain fatty acids positively influenced both sow and piglet performance during the lactation period as well as the continued supplementation on post-weaning phase. Thus, after 25 days of supplementation, sows experienced reduced fat reserve depletion compared to the control group, suggesting enhanced nutrient metabolism with potential metabolite transfer to their offspring. This was evident in piglets nursed by supplemented sows, which weighed 320 g more at weaning and exhibited greater weight uniformity compared to those in the control group.

The primary hypothesis of this research was that supplementing sows’ diets with SCFAs-MCFAs during late gestation and lactation would influence offspring’s intestinal microbial composition, potentially enhancing pig performance during the critical pre-weaning period. Piglets are born with immature digestive and immune systems, making them highly susceptible to various pathologies, most commonly of an infectious nature. Previous research in both animal and human medicine has indicated that early microbial colonization can be a powerful strategy to support the development of the newborn’s immune system, enhance growth, and improve survival rates, with lasting positive effects [13,18,19,20]. Although the exact timing of microbiota development in newborns is not fully understood, it is evident that it is influenced by the microbiota of both the mother and the surrounding environment. During birth, piglets are exposed to the maternal microbiota as they pass through the birth canal, which serves as their primary source of microbial inoculation. This initial exposure is further shaped during lactation as piglets ingest milk containing maternal microbiota and remain in constant contact with the sow’s skin, feces, and urine. Therefore, the sow plays a pivotal role in establishing a diverse and stable gut microbial community, which greatly impacts the health and feed intake of suckling piglets [21,22,23]. In this study, after 7 days of lactation, sows fed a diet supplemented with SCFAs-MCFAs exhibited a reduced *Streptococcus suis* population compared to those on the control diet. Interestingly, this reduction in *S. suis* was not observed in the feces of suckling piglets, although they showed a higher count of lactic acid bacteria. It is worth noting that changes in the piglet’s microbial composition were more pronounced at the end of the lactation period (day 20). Nowland et al. [22] have suggested that specific microbial changes in the sow’s gastrointestinal tract (GIT) are not always mirrored in their offspring. Similarly, a study by Berry et al. [24] found *Lactobacillus* and *Clostridium* present in piglet feces even when these bacteria were absent from the sow’s feces. In a comparable evaluation, *C. perfringens* counts were significantly reduced in sows fed organic acids, but no corresponding difference was noted in piglet microbial counts [13]. In contrast, Devi and colleagues [11] observed a reduction in *E. coli* concentrations in both sows and their weaning piglets following organic acid supplementation during lactation. The present study’s findings suggest that the positive modulation of piglet microbiota at the end of the lactation period could be due to progressive colonization resulting from early exposure to beneficial bacteria shared by their mothers. This is particularly relevant as piglets remained exclusively with their sow in individual pens throughout lactation.

In addition to the maternal and environmental microbial modulation, the positive effect on litter performance (320 g higher than control litters) can likely be attributed to the synergistic effects of SCFAs and MCFAs in the sow’s diet. These compounds probably enhanced sow digestion, thereby alleviating the negative energy balance often observed in lactating sows and supporting effective nursing and piglet growth. Physiologically, lactating sows face high energy demands, with milk production requiring approximately 14-18 MJ/day, depending on litter size and piglet growth rates [16]. Insufficient dietary energy intake often results in the mobilization of body reserves, leading to backfat loss and potentially impairing subsequent reproductive performance, particularly in highly prolific modern sows [25]. In this context, the addition of SCFAs and MCFAs may have contributed to this improved performance through several mechanisms, including the following: (1) lowering stomach pH; (2) stimulating digestive enzyme activity (e.g., activating pepsinogen to pepsin); (3) suppressing the proliferation of pathogenic bacteria; (4) enhancing gut morphology; (5) improving nutrient retention; (6) optimizing mineral and energy utilization [5,10,26,27]. Additionally, SCFAs have been suggested to serve as energy substrates through gluconeogenesis or direct oxidation in the liver, while MCFAs are efficiently metabolized via β-oxidation, bypassing the lymphatic system [28]. Thus, previous studies have related organic acids with anti-agalactia properties [29], increased backfat thickness, and increased fat content in milk [30,31]. Given these promising results, further research is strongly recommended to incorporate measurements of milk fat content and sow nutrient digestibility to better understand and quantify the mechanisms driving these improvements.

The second hypothesis proposed that the post-weaning performance of piglets nursed by sows supplemented with SCFAs-MCFAs would be superior to those nursed by non-supplemented sows, with further benefits seen when SCFAs-MCFAs feed was added to post-weaning feed. Previous research on maternal diets with supplements such as conjugated linoleic acid [32,33], seaweed extracts, and fish oil supplementation [34] and nutrient deprivation [35] has demonstrated their potential impact on growth performance, immune function, and gastrointestinal health in suckling, weanling, and post-weaning pigs.

In this study, maternal diet notably influenced fecal microbial composition for the first 7 days post-weaning. Pigs weaned from SCFAs-MCFAs-supplemented sows but fed a control diet exhibited the lowest *C. perfringens* counts and higher lactic acid bacteria counts, suggesting an early-life establishment of beneficial microbiota, as previously discussed. Surprisingly, despite this positive microbial modulation, there was no long-term effect of maternal feed on post-weaning pig performance, nor an interaction with piglet feed, even though pre-weaning litter performance was superior under the SCFAs-MCFAs diet. One explanation for the lack of sustained effects could be the profound microbial shifts that occur once sow milk is no longer available, typically within the first 7 to 14 days after weaning [36]. Additionally, the body weight homogenization performed before the experiment to standardize starting conditions may have masked potential differences in post-weaning performance. Moreover, the commercial environment, colonized by a diverse range of microorganisms, likely influenced the microbial composition of newly weaned piglets. It is important to highlight that this is a multifaceted process influenced by various factors, including the environmental microbial load, dietary transitions, and the inherent variability of individual microbiota. At this point, the new feed provided in the weaning unit was expected to have a major influence in shaping the total population and diversity of the intestinal microbiota. It is known that the transition from milk to solid feed introduces significant dietary changes that profoundly shape the microbial community in the gastrointestinal tract. Indeed, specialized SCFAs-MCFAs supplementation in post-weaning diets did influence intestinal bacterial composition, resulting in reduced *S. suis* and *E. coli* counts, along with a reduction in lactic acid bacteria but an increase in *C. perfringens* counts after 7 days of feeding. This effect persisted through 35 days of supplementation. Although there was a decrease in lactic acid bacteria counts, the significant reduction in pathogenic bacteria, particularly *E. coli* and *S. suis*, likely contributed to improved pig performance, especially in terms of feed efficiency observed 14 days post-weaning. Unlike mature pigs, which maintain a gastric pH range of 2.0 to 3.0, the gastric pH in suckling and weaned piglets tends to be higher, ranging between 2.6 and 5.0. Maintaining a low gastric pH is crucial not only for preventing pathogen proliferation but also for optimizing nutrient digestion (e.g., pepsin enzyme activation) and absorption [37]. Several studies have investigated the impact of incorporating SCFAs such as lactic, formic, and propionic acid into pig diets to lower the pH of the intestinal digesta, aiming to suppress the overgrowth of pathogenic bacteria and mitigate post-weaning diarrhea in piglets [38]. Their primary site of action is the proximal gastrointestinal tract, including the stomach and small intestine, where their antibacterial properties are vital for reducing the risk of *E. coli* infections. Thus, SCFAs have the ability to penetrate bacterial cell membranes and dissociate within the cell, releasing protons and anions that disrupt intracellular pH balance. In conditions of a more neutral pH, dissociation of the acids leads to the production of toxic anions and protons, which interfere with bacterial energy production by altering the transmembrane proton gradient [37]. Elevated cytoplasmic pH can be fatal to bacteria, affecting purine integrity and denaturing critical enzymes. Regarding, MCFAs also exhibit strong antibacterial activity by crossing bacterial membranes and damaging their internal structures [39]. Studies have highlighted the effects of specific MCFAs, such as caprylic (C8:0) and capric acids (C10:0), on both Gram-positive and Gram-negative bacteria in vitro [40]. Furthermore, the combined use of SCFAs and MCFAs (0.2% to 0.4%) to pigs challenged with *E. coli* around weaning significantly reduced diarrhea incidence [41]. Indeed, the growth response to organic acid supplementation has been shown to be more pronounced in weaning pigs compared to older animals. For example, a meta-analysis reported growth rate improvements of 12.25% and 6.03% during the first 2 and 4 weeks post-weaning, respectively, while the benefits were lower in growing (3.51%) and finishing (2.69%) pigs [37]. These differences can be attributed to several factors, including the type and dose of organic acids used, duration of supplementation, dietary composition and buffering capacity, hygiene and welfare conditions, animal health status, and age [37]. Indeed, one of the key qualities influencing antimicrobial activity is a pKa between 3 and 5, which enhances the efficacy of organic acids [42]. Thus, MCFAs primarily targeted Gram-positive bacteria (e.g., *C. perfringens*, *Enterococcus* spp., *Streptococcus* spp.), while SCFAs exhibited greater efficacy against Gram-negative bacteria (e.g., *E. coli*, *Campylobacter jejuni*, *Salmonella* spp.) [37]. This dual action, used in the present study, underscores the potential of these compounds to improve gut health and performance in weaned pigs. Extensive research in the swine industry has consistently shown a strong correlation between intestinal microbiota and animal performance [5,21,43,44,45,46], emphasizing the potential for microbiota manipulation to enhance health and feed efficiency.

A more detailed analysis of microbial populations, incorporating techniques such as 16S rRNA sequencing, alongside the evaluation of immune markers and nutrient digestibility, could have offered deeper insights into the specific bacterial patterns affected by the antimicrobial properties of organic acids. These insights would help elucidate the mechanisms underlying the improved feed efficiency observed in this study.

## 5. Conclusions

In conclusion, supplementing maternal diets with synergistic blend of SCFAs-MCFAs influences early microbial colonization in piglets by modulating the fragile gut environment after parturition, with effects potentially lasting into the early post-weaning period. This supplementation partially helps alleviate the negative energy balance in lactating sows, supporting effective nursing and piglet growth. The specialized combination of SCFAs-MCFAs in post-weaning feed further enhances piglet growth, reduces pathogenic bacteria, and improves feed efficiency. These findings are particularly valuable in intensive farming, where promoting pig health, ensuring uniform growth, and reducing antibiotic use are priorities. Further research on microbial populations, immune markers, and nutrient digestibility is recommended to clarify the mechanisms driving these benefits.

## Figures and Tables

**Table 1:** Ingredient and nutrient composition of the basal lactation diet, on an as-fed basis.

Ingredients, %	Lactation
Corn	21.51
Wheat	15.00
Wheat bran	15.00
Field pea	12.11
Soybean	11.75
Rice bran	10.00
Sorghum	5.00
Sugarbeet pulp	5.00
Calcium carbonate	1.66
Palm oil	1.06
Lysine 50	0.30
L-Threonine	0.10
DL-Methionine	0.06
Salt	0.60
Mono calcium phosphate	0.43
Vit-Min Premix ^1^	0.40
Phytase	0.02
Calculated composition	
NE, kcal/kg	2230
CP, %	15.94
Lysine, %	0.94
Ether extract, %	4.50
Crude fiber, %	5.08
Ca, %	0.85
Total P, %	0.62
Dig P, %	0.30
Analyzed nutrient composition, %	
CP	15.90
Ether Extract	4.00
NDF	16.49
Ash	5.94

^1^ The following amounts were provided per kg diet: 2500 IU vitamin A (acetate); 500 IU 25-hydroxy vitamin D3 (HyD, DSM Nutritional Products, Basel, Switzerland); 43.75 mg DL-alphatocoferol; 625 mg vitamin B1; 1.75 g vitamin B2; 750 mg vitamin B6; 8.75 mg vitamin B12; 150 mg biotin; 2.50 g D-pantothenic calcium; 7.50 g niacin; 2 g folic acid; 1 g vitamin K3; 50 g choline chloride; 37.5 mg Fe (FeSO_4_); 15 mg Mn (MnO); 2.5 mg Cu (CuSO_4_.5H_2_O); 25 mg Zn (ZnO); 500 mg I (KI); 100 mg Se (Na_2_SeO_3_).

**Table 2:** Ingredient and nutrient composition of the basal pig diet, on an as-fed basis.

Ingredients, %	Pre-Starter	Starter
Wheat	33.40	30.27
Barley Extruded	30.00	10.00
Soybean meal, 47	7.79	10.91
Corn	-	20.00
Sorghum	-	15.00
Dextrose	7.50	-
Whey powder acid	5.00	-
Fish meal, 70	5.00	2.50
Mucous hydrolyzed, 50	-	2.50
Animal plasma, 80	2.50	-
Soybean concentrate	2.00	2.50
Rapeseed oil	1.20	-
Bi-calcium phosphate	1.00	-
Lard 1.5	-	1.68
Lysine 50	-	0.95
Calcium carbonate	-	0.90
Monocalcium phosphate	-	0.70
Vitamin Premix ^1^	2.50	1.00
Lysine sulphate 70	0.85	-
DL-Methionine	0.32	0.21
L-Threonine	0.32	0.26
Salt	0.20	0.30
L-Tryptophan	0.12	0.06
L-Valine	0.12	0.08
Enzymes	0.10	0.10
Phytase	0.08	0.08
Calculated composition		
NE, kcal/kg	2430	2380
CP, %	17.62	17.47
Lysine, %	1.39	1.29
Ether extract, %	4.50	3.91
Crude fiber, %	2.86	2.58
Ca, %	0.52	0.6
Total P, %	0.62	0.54
Dig P, %	0.73	0.67
Analyzed nutrient composition, %		
CP	18.54	17.28
Ether Extract	4.58	4.20
NDF	10.53	12.37
Ash	5.77	5.56

^1^ The following amounts were provided per kg of diet: 1000 IU vitamin A (acetate); 2000 IU 25-hydroxy vitamin D3 (HyD, DSM Nutritional Products, Basel, Switzerland); 10 IU DL-alphatocoferol; 1 mg vitamin B1; 2.7 mg vitamin B2; 1.8 mg vitamin B6; 0.03 mg vitamin B12; 11 mg D-pantothenic acid; 15 mg niacin; 99 mg iron (FeSO_4_); 90 mg Cu (Glycine hydrate chelate); 199 mg iodine (KI); 19.8 mg selenium (Na_2_SeO_3_); 600 mg butylhydroxytoluene.

**Table 3:** Productive performance of lactating sows fed control (MCON) and SCFAs-MCFAs (MOA) diet.

Item	MCON	MOA	SEM	*p*-Value
Nº of sows	36	36	-	-
Parity, n	4.6	4.9	0.18	0.251
Sow BW, kg				
Gestation (day 110)	266.2	267.8	16.64	0.783
Weaning	223.5	226.5	18.81	0.551
Sow backfat, mm				
Gestation (day 110)	17.2	17.1	0.79	0.942
Weaning	13.4	14.2	0.65	0.215
Loss backfat, mm	−3.73	−2.88	0.28	0.023
Feed intake, kg				
Total Feed intake	117.7	124.0	6.96	0.268
ADFI	4.6	4.9	0.26	0.268
Weaning-estrus interval, d	4.9	4.8	0.30	0.943

**Table 4:** Reproductive performance of lactating sows fed control (MCON) and SCFAs-MCFAs (MOA) diets.

Item	MCON	MOA	SEM	*p*-Value
Litter birth weight, kg	19.18	18.85	0.601	0.673
Born alive pig weight, kg	1.40	1.38	0.042	0.695
Total born pigs ^1^, n	14.36	14.66	0.482	0.574
Pigs born alive, n	13.20	12.91	0.527	0.523
Pigs stillborn, n	1.03	1.35	0.227	0.307
Suckling litter performance ^2^				
Litter size at cross-fostering (CF), n	12.62	12.24	0.165	0.109
Pig CF weight, kg	1.71	1.74	0.048	0.612
Litter performance day 20 lactation ^3^				
Litter weight, kg	65.30	65.78	1.839	0.844
Pig weight, kg	5.65	5.97	0.101	0.027
Total weaned pigs, n	11.58	11.08	0.245	0.140
CV, %	18.56	15.88	0.833	0.024

^1^ Total born includes born alive, stillborn and mummified pigs. ^2^ Litter sizes were adjusted by cross-fostering within treatment between 24 and 48 h after farrowing. ^3^ Piglet BW at cross-fostering was used as covariable.

**Table 5:** Growth performance of weaned pigs as affected by dietary control (MCON) or SCFAs-MCFAs supplementation (MOA) to sows during lactation and post-weaning diets without (CON) and with specialized SCFAs-MCFAs supplementation (OA) ^1^.

Sow Diet	Pig Diet	BW, kg			ADFI, g/d			ADG, g/d			G:F		
		d 0	d 14	d 35	d 0–14	d 14–35	d 0–35	d 0–14	d 14–35	d 0–35	d 0–14	d 14–35	d 0–35
MCON		6.20	8.10	15.29	151.0	409.9	306.4	141.2	342.0	261.9	0.92	0.84	0.86
MOA		6.19	8.10	15.20	152.8	403.8	301.5	136.5	338.0	257.6	0.89	0.84	0.86
SEM		0.632	0.725	1.116	6.641	25.558	16.667	6.410	21.663	13.979	0.023	0.053	0.036
	CON	6.19	8.02	15.07	150.5	406.4	303.6	135.4	335.9	255.9	0.89	0.83	0.85
	OA	6.19	8.19	15.42	153.3	407.3	304.3	142.3	344.1	263.6	0.93	0.85	0.87
SEM		0.632	0.725	1.116	6.641	25.558	16.667	6.410	21.639	13.979	0.023	0.053	0.040
*p*-value													
Sow diet		0.309	0.993	0.647	0.579	0.532	0.372	0.305	0.557	0.367	0.194	0.919	0.977
Pig diet		0.926	0.099	0.068	0.373	0.927	0.900	0.136	0.236	0.113	0.050	0.373	0.233
Sow × pig diet		0.602	0.310	0.315	0.779	0.857	0.603	0.878	0.571	0.530	0.176	0.589	0.421

^1^ Data are means of 12 replicate pens for the two-way interaction. Two-way interaction was not significant for any growth performance parameter; therefore, data were reanalyzed for main factors. Data are means of 24 replicate pens (11 pigs per replicate pen) per main factors of sow and pig diet.

**Table 6:** Plating microbial count from pigs as affected by dietary control (MCON) or SCFAs-MCFAs supplementation (MOA) to sows during lactation and post-weaning diets without (CON) and with specialized SCFAs-MCFAs supplementation (OA) at 7 and 35 days post-weaning ^1^.

Sow Diet	Pig Diet	7 d Post-Weaning				35 d Post-Weaning			
		*S. suis*	*E. coli*	*C. perfringens*	*Lactic acid bacteria*	*S. suis*	*E. coli*	*C. perfringens*	*Lactic acid bacteria*
MCON	CON	3.42	7.97	3.65 ^a^	8.05 ^a^	5.36	7.21 ^a^	3.14	7.85
	OA	2.97	7.58	3.62 ^a^	7.82 ^b^	5.22	6.66 ^b^	3.23	7.34
MOA	CON	3.21	7.80	3.34 ^b^	8.02 ^a^	5.24	6.86 ^ab^	3.22	7.43
	OA	2.98	7.37	3.60 ^a^	8.02 ^a^	5.30	6.96 ^ab^	3.43	7.22
SEM		0.112	0.215	0.060	0.060	0.138	0.144	0.104	0.274
*p*-value									
Sow diet		0.372	0.216	0.0001	0.035	0.820	0.837	0.067	0.161
Pig diet		0.003	0.008	0.009	0.007	0.675	0.028	0.038	0.065
Sow × pig diet		0.344	0.910	0.001	0.008	0.321	0.002	0.448	0.435

^1^ Data are means of 3 pigs per 12 replicate pens for the two-way interaction. ^a, b^ Values within the same column with different letters differ significantly (*p* < 0.05).

## Data Availability

The data presented in this study are available on request from the corresponding author. The data are not publicly available due to confidentiality between the contributed parties.
